# The effect of health financing reforms on incidence and management of childhood infections in Ghana: a matching difference in differences impact evaluation

**DOI:** 10.1186/s12889-022-13934-y

**Published:** 2022-08-05

**Authors:** Emmanuel Nene Odjidja, Ruth Ansah-Akrofi, Arnaud Iradukunda, Charles Kwanin, Manika Saha

**Affiliations:** 1 Department of Monitoring and Evaluation, Kigutu Village Health Works, Kirungu, Burundi; 2 Department of Statistics and Computer Science, University of Ghana, Accra, Ghana; 3 Department of Medicine, University of Burundi, Bujumbura, Burundi; 4 Ghana Health Service, University of Geneva, Geneva, Switzerland; 5 Faculty of Information Technology, Monash University, Melbourne, Australia

**Keywords:** Health Insurance, Impact evaluation, Propensity score matching, Difference in differences, Ghana, Sub-Saharan Africa

## Abstract

**Introduction:**

In 2003, Ghana abolished direct out of pockets payments and implemented health financing reforms including the national health insurance scheme in 2004. Treatment of childhood infections is a key component of services covered under this scheme, yet, outcomes on incidence and treatment of these infections after introducing these reforms have not been covered in evaluation studies. This study fills this gap by assessing the impact on the reforms on the two most dominant childhood infections; fever (malaria) and diarrhoea.

**Methods:**

Nigeria was used as the control country with pre-intervention period of 1990 and 2003 and 1993 and 1998 in Ghana. Post-intervention period was 2008 and 2014 in Ghana and 2008 and 2018 in Nigeria. Data was acquired from demographic health surveys in both countries and propensity score matching was calculated based on background socioeconomic covariates. Following matching, difference in difference analysis was conducted to estimate average treatment on the treated effects. All analysis were conducted in STATA (psmatch2, psgraph and pstest) and statistical significance was considered when *p*-value ≤ 0.05.

**Results:**

After matching, it was determined that health reforms significantly increased general medical care for children with diarrhoea (25 percentage points) and fever (40 percentage points). Also for those receiving care specifically in government managed facilities for diarrhoea (14 percentage points) and fever (24 percentage points).

**Conclusions:**

Introduction of health financing reforms in Ghana had positive effects on childhood infections (malaria and diarrhoea).

## Introduction

Removal of financial barriers to health access is fundamental to the achievement of universal health coverage [1]. Universal health coverage has been recognised as the single most important equitable public health intervention aimed at protecting the poor and vulnerable from catastrophic costs when accessing healthcare [2]. In recent times, developing countries, including those in sub-Saharan Africa, have embarked on reforms in increasing health access via different financing schemes notably social health insurance schemes [3]. While the underlying rationale of these insurance schemes are meant to protect the poorest and vulnerable from catastrophic health costs, its design and implementation have varied across countries and regions [4]. From Rwanda’s Mutuelle de Santé to Ghana’s national health insurance scheme (NHIS), countries have not only implemented different mechanisms but have also targeted diverse populations and offered varied services under these schemes [5].

In 2003, Ghana passed the NHIS Act 650, removing financial barriers to access to essential health services [6]. Services covered under this scheme include management and treatment of childhood infections [6]. With an Under-5 mortality rate of 47.9 deaths per 1000 live births, childhood infections including malaria and diarrhoea are among the significant factors of under-five deaths, contributing 60% to all causes of deaths [7]. According to the Demographic Health Survey (DHS), between 2003 and 2014, the incidence of febrile illnesses among children decreased from 21.3% to 13.8% and diarrhoea incidence also reduced from 15.2% to 11.7%. This improvement also followed marginal increases in the percentage of caregivers seeking care for children with both illnesses during the same period. Despite this, there are no impact evaluations assessing the possible link between recent financial reforms and increases in health access for the treatment of these childhood infections.

Previous nationwide evaluations of the NHIS have mainly focused on maternal health care utilization and to a lesser extent, access to infant health care. Bonfrer et al. [8] focused on access to antenatal care, deliveries and infant vaccination. Leone et al. [9] on the other hand analysed the effect of the health reforms on facility deliveries and caesarean section and a study by Blanchet et al. [10] only focused narrowly on aspects of hospitalization and out-patient services without an assessment of maternal health. Furthermore, methodologically, these impact evaluation studies have either employed propensity score matching or difference in differences [8–10]. However, evidence has increasingly established that using either of these methods could result in bias leading to errors in impact estimates. Therefore, a major recommendation to this challenge has been combining two or more different quasi-experimental techniques [11].

This study addresses the gaps mentioned above via combining propensity score matching and difference in difference analysis to estimate the impact of health financial reforms on the incidence and access to treatment for childhood infections.

## Methods

### Data sources

Data for all analysis presented in this study were acquired from the Ghana and Nigeria Demographic Health Survey; a, a nationwide representative survey held every five years in developing countries [12]. Responses of outcomes assessed were acquired from a verbal recall of caregivers, who were mainly mothers of infants.

Given that the financial reforms were mainly introduced in 2003, we selected four surveys, two before the policy introduction and other two after implementation as recommended by Leone et al. [9]. To improve the level of analysis we selected surveys in the early 1990s to late 1990s and early 2000s as pre-intervention periods. Post-intervention surveys were selected between 2008 and 2018.

Nigeria was considered as the comparison country (to Ghana) for this study as there was no clear federal level targeting fees removal for child health services during the study period [13]. In spite of this, some states had piloted and implemented fee exemption for minimum packages for maternal and child health services. For example, the free maternal and child healthcare programme piloted in Enugu in 2007 [14]. The Nigeria Demographic Health Survey (NDHS) data for 1990 and 2003 was used as the pre-intervention period whereas data 2008 and 2018 was used as comparative post-intervention period [12]. Ghana was the treatment country as the implementation of the National Health Insurance scheme was nationwide and access was unrestricted. The pre and post intervention period for Ghana was 1993, 1998 and 2008, 2014 respectively.

### Study design

The original study was a cross-sectional study, however, this secondary analysis is a quasi-experimental impact evaluation, employing both propensity score matching and difference in differences.

### Outcomes under study

Eight outcomes pertaining to incidence and management of diarrhoea and fever were selected for this study; the incidence of fever (a proxy for malaria), medical treatment for diarrhoea, medical treatment for diarrhoea in a health facility owned by the government, given oral rehydration for treatment of diarrhoea, fever incidence, medical treatment for fever, care for fever in a health facility owned by the government and given antimalarial treatment. All outcomes were defined in line with the definition offered in the guide to the DHS statistics.

Diarrhoea and fever incidence had a binary response (Yes/No) as to whether any child under age five had any of the two illnesses two weeks preceding the date of survey [15].

Medical care for diarrhoea and fever is also a binary variable, and it was defined as the number of children with either illness, receiving medical advice from allopathic health sources irrespective of whether it is owned by a private or public entity [15]. Those receiving medical care specifically from health facilities owned by the government was considered as an outcome as most financial reforms were initially implemented in those facilities recent expansion to the private sector.

Children receiving oral rehydration, a binary variable, was defined as children with diarrhoea two weeks preceding the survey which sought medical care and were given any form of oral rehydration as part of treatment. Likewise, those given antimalarial as part of medical treatment was defined as children with fever two weeks preceding the survey and sought medical treatment and received any type of antimalarial.

### Statistical analysis

Given that health financing reforms were nationwide, far reaching all significant parts of the health systems, we considered caregivers in Ghana as receiving the intervention and matched with untreated based on selected covariates in Nigeria.

Having first been developed by Rosenbaum & Rubin [16], propensity scores predict the probability of receiving a treatment given selected covariates. To estimate propensity scores in this study, we selected a varied range of covariates from educational to socioeconomic backgrounds. Specifically, variables used to estimate propensity score included binary variables “radio ownership by household (yes/no)”, “place of residence of child (rural/urban)”, highest education level of caregiver (secondary or above/lower)”, “source of drinking water of household (improved/unimproved)” and “type of toilet facility of household (improved/unimproved)”. Another variable included was the age of child under 5 within the household. Then, using a probit regression model, we predict the probability of intervention assignment to acquire the propensity scores.

To match intervention observations with untreated, we select a kernel matching technique, emphasizing on observations that fell within the area of common support. Kernel matching is preferred to one-on-one matching as it offers better matching controls [17]. To reduce the possible bias emanating from the ex-post effect of the intervention, we match observations based on pre-intervention background characteristics.

Quality of post matching balance was assessed using mean differences between intervention arms and matching controls along with the percentage of bias, t test with *p*-value and variance ratios [18]. As recommended by Rubin [19], a substantial imbalance was flagged when the percentage of bias (via the mean difference) was above 0.1 and the variance ratio fell within the ranges of 0.8 and 1.25.

The second stage statistical analysis involved estimating the average treatment on the treated effects (ATT) using a difference in difference modelling. Pre-intervention trends were compared to post-intervention trends between the matched treated and untreated. For pragmatic reasons of interpretation, a linear probability model instead of a logit or probit model was modelled to estimate impact. This was denoted as:

1 $$Y_i=\alpha+\beta T_i+\gamma t_i+\delta\left(T_i\ast t_i\right)+\varepsilon_i$$

where α = the constant variable.

β = specific effect ascribed to the intervention group.

γ = time trend which is same between intervention and untreated groups.

δ = the true effect, which is an interaction between the difference in outcome between treatment and untreated given the pre and post-intervention trends.

All analysis were conducted in STATA 13.0, specifically, the “*psmatch2*” package was used to create propensity scores and matching along with the “*pstest*” and “*psgraph*” to test the balancing property and graph results of the balancing respectively. The difference in differences was done by using the command “diff”. The sample size post matching was sufficient, therefore, no bootstrapping techniques was necessitated. Statistical significance was considered when *p*-value ≤ 0.05.

## Results

Overall, hitherto matching, pre-intervention observations were 19,433 with 28.3% in the treatment arm. Post-intervention observations increased to 71,447, of which, the unmatched treated arm comprised 12.4%. However, following propensity score matching techniques, the pre-intervention reduced to 22,717, consisting of 25.8% of those treated (Table 1). The area of common support was demarcated between a variance ratio region of 0.92 and 1.09. By these criteria, all matched treated, and untreated observations fell within the area of common support (Table 1).

**Table 1:** Matching Assignment of observations by area of common support

Psmatch2: Treatment Assignment	Psmatch2: Common Support		Total
	Off support	On support	
Untreated	0	16,862	16,862
Treated	0	5,855	5,855
Total	0	22,717	22,7717

As shown in Table 2, all covariates except the source of drinking water and television ownership were significantly associated with treatment. Type of toilet facility, although associated with treatment, showed imbalance between treated and untreated during the test for balancing property, therefore, it was dropped.

**Table 2:** Probit Regression predicting probability of treatment

Variable	Coefficient/SE	*P*-value
Has Radio	0.130 (0.011)	< 0.001
Highest Educational Level	0.136 (0.006)	< 0.001
Age of under-5 children	0.037 (0.003)	< 0.001
Place of residence	0.037 (0.012)	< 0.001
Source of drinking water	-0.013 (0.003)	< 0.001
Type of toilet facility	0.006 (0.006)	< 0.001
Has Television	-0.160 (0.137)	< 0.001

Among matched observations, mean of those with radio, a binary economic indicator was slightly higher among the treated (0.03%), but this resulted in 0.0% bias and was statistically insignificant (*p* = 0.987). Furthermore, the level of education was slightly higher among the treated with a mean difference of 0.00052 (Table 3). Mean age of children under five assessed between matched treated and untreated was 3.6663 and 3.6668, respectively (*p* = 0.985). Place of residence, coded as 1 and 2 for rural and urban settings, was the same for both matched treatment and control.

**Table 3:** Standardized differences between treatment and control with balancing property

Covariate Variables	Mean			T-test		Var(T)/Var (C)
	Treated	Control	%bias	t	*p*-value	
Has radio	0.54347	0.5433	0.0	0.02	0.987	1.00
Highest Level of Education	0.93271	0.93219	0.1	0.03	0.975	1.00
Age of under-5 children	3.6663	3.6668	-0.0	-0.02	0.985	1.00
Place of residence	1.719	1.719	0.0	-0.00	1.000	1.00

**Fig. 1:**
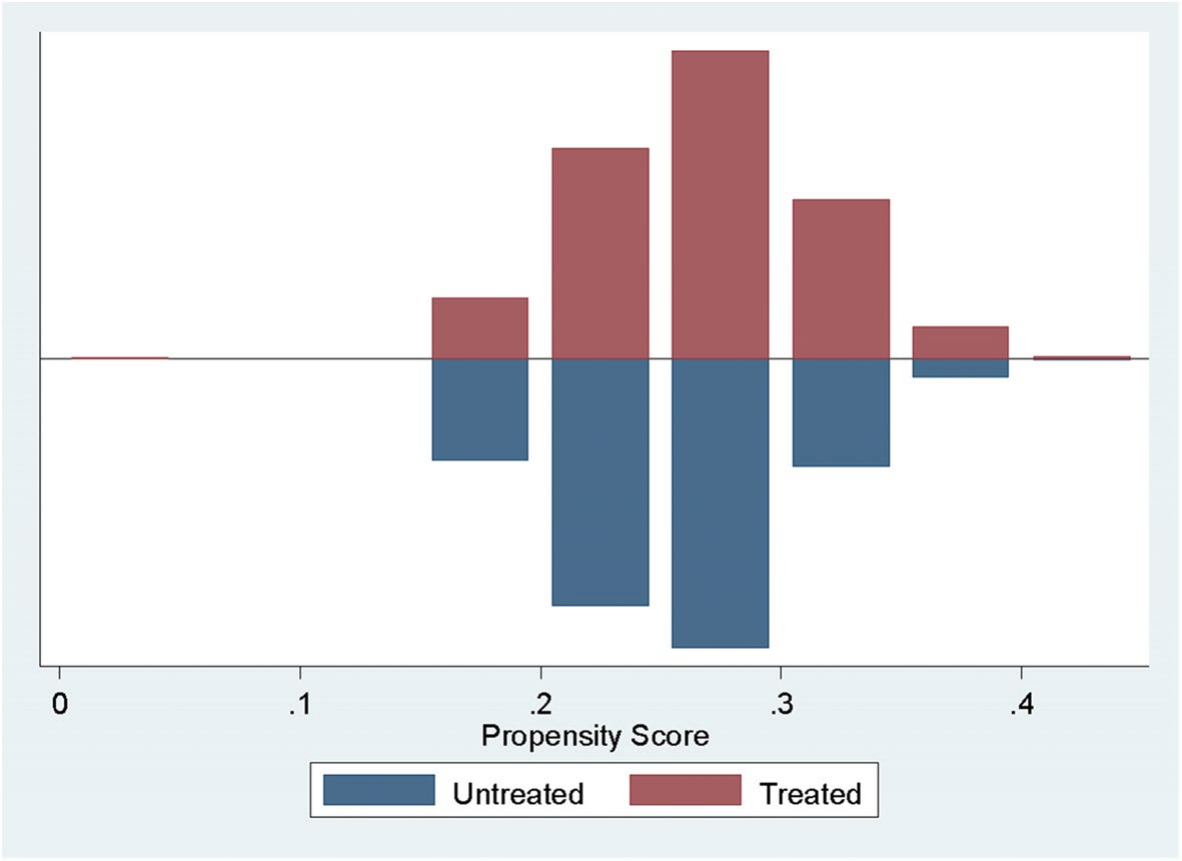
Propensity Scores between matched treated and untreated

Employing the conditions of balancing stated in the methodology, all covariates were appropriate and satisfied the balancing property as graphically shown in Fig. 1.

### Effects of financial reforms on diarrhoea incidence and management

Following the balancing test between treatment and untreated, economic reforms introduced after 2003 in Ghana increased the proportion of children with diarrhoea seeking care by 25.4 percentage points (*p* < 0.01) and also increased the proportion of those who received care from government-owned facilities (14.7 pp, *p* < 0.01). No effect was estimated on the incidence of diarrhoea (*p* = 0.250) and proportion of children with diarrhoea who received oral rehydration as part of medical treatment (*p* = 0.778) (Table 4).

**Table 4:** Difference in differences coefficients and standard error using linear probability models

Outcome Variable	ATT/SE	*P*-value
Diarrhoea Incidence	-0.021 (0.018)	0.250
Medical care for children with diarrhoea	0.254 (0.039)	< 0.01
Received medical care for diarrhoea in a government owned health facility	0.147 (0.022)	< 0.01
Received oral rehydration for diarrhoea treatment	-0.014 (0.049)	0.778

### Effects of financial reforms on fever (a proxy for malaria) incidence and management

Although fever incidence increased significantly between pre and post-intervention periods, those who sought medical care increased by 40 percentage points (*p* < 0.01). Furthermore, average treatment on the treated (ATT) effects was significant for the proportion of children with fever who sought care (24.1 pp, *p* < 0.01). Health financing reforms in Ghana (in comparison to Nigeria) significantly increased the proportion of children who received antimalarial treatment as part of routine treatment for fever by 5.3 percentage points (*p* = 0.01) (Table 5).

**Table 5:** Difference in differences coefficients and standard error using linear probability models

Outcome Variable	ATT/SE	*P*-value
Fever (Malaria) Incidence	0.399 (0.025)	< 0.01
Medical care for children with fever	0.400 (0.033)	< 0.01
Received medical care for fever in a government-owned health facility	0.241 (0.020)	< 0.01
Received antimalarial for fever (malaria) treatment	0.053 (0.015)	0.01

## Discussion

This study combined two quasi-experimental methods; propensity score matching and difference in difference to estimate average treatment effects of health financing reforms on incidence and access to treatment of childhood infections. To our knowledge, this is the first study that adopts two quasi-experimental methods to assess the effects of these reforms on childhood infections in the Ghanaian context. We found significant average treatment on the treated impacts for children with diarrhoea seeking medical care and those who received care from government-owned facilities. Trend of incidence on diarrhoeal disease was not significantly affected following the introduction of health reforms in Ghana. Since its inception, the NHIS has included treatment of diarrhoea as part of minimum services [20] and this has yielded some positive gains. Between 2003 and 2014, access to diarrhoea medical care increased by 31.8% [12]. In addition to these health financing reforms, other reforms such as sector-wide prioritization also set access to care for childhood infections a key priority. These priorities have also informed the strategies of donor organisations and global health partners. For example, the implementation of different initiatives in increasing medical care for diarrhoea under the Strengthening Health Outcomes through the Private Sector (SHOPS) project funded by USAID [21].

Average treatment effects on malaria incidence was 39.9% percentage points (< 0.01), indicating that financial reforms may have an increased incidence of malaria. Studies have increasingly established that removal of direct payments may encourage positive changes in health-seeking behaviours, thereby, increasing the rate of disease incidence [22]. In Ansah and colleagues’ study [22], the removal of financial barriers at primary healthcare increased detection of anaemia by 3.2% in the intervention arm. In our study, an increase in fever incidence was also followed by significant ATT for all indicators of health access and medication, including antimalarial treatment. Like diarrhoea treatment, access to malaria treatment was prioritised under the NHIS, reducing catastrophic health expenses at the household level. Dalaba et al. [23] found that insured households under the NHIS reduced direct health costs by 21.1% and on overall, reduced direct and indirect health costs by 9.70%. In malaria-endemic areas where the risk of recurrent malarial infections is imminent, such financial cover could have significant benefits to caregivers, especially those in the poorest households.

This study is subject to several limitations. First, the small number of covariates used in calculating the propensity scores could have resulted in selection bias in matching untreated observations to those treated. However, in the quest to ensure consistency between Nigeria and Ghana dataset for the study duration, we only selected covariates that remained constant with the same interpretation between both countries. This resulted in a narrow spectrum of covariates selected, all of whom were strongly associated with treatment assignment. Also, as shown by Thavaneswaran and Lix [24], there is no recommended rule on the optimal number of covariates for propensity score computation. Secondly, the primary outcome data was collected via verbal recall on account of the caregiver. While this could have probably resulted in underestimation or overestimation of results, this approach as employed by the DHS has validated in many settings and has been found to offer accurate estimates [25]. Thirdly, it should be noted that these variables are not exhaustive in determining child health status. Thus, there are other contextual environmental, social, economic cofounders which have not been examined in this study.

## Conclusion

Through the use of quasi-experimental methods, we found that the introduction of the NHIS had positive effects on access to medical care for malaria and diarrhoea but not diarrhoea incidence and access to oral rehydration therapy. Access to antimalarial treatment was also significantly impacted following the introduction of these programs.

While findings of this study suggest that health reforms such as insurance could be beneficial to child health, indirect health costs could pose a challenge to access and this will disproportionately affect the poor. However, this study did not assess the impact of these reforms on the poor and other vulnerable groups due to the data limitations. Subsequent studies should combine other robust methods, including qualitative ones, to fill this gap.

## Data Availability

The datasets generated and/or analysed during the current study are available in the MEASURE DHS website: http://dhsprogram.com/data/availabledatasets.cfm.

## Abbreviations

ATT: Average Treatment on Treated
DHS: Demographic Health Survey
NDHS: Nigeria Demographic Health Survey
NHIS: National Health Insurance Scheme
SHOPS: Strengthening Health Outcomes through the Private Sector
USAID: United States Agency for International Development
